# Electroacupuncture Alleviates Hyperalgesia and Anxiety-Like Behaviors in Pain Memory Model Rats Through Activation of GABAergic Neurons and GABA Receptor in the Rostral Anterior Cingulate Cortex

**DOI:** 10.1007/s12035-024-03986-z

**Published:** 2024-02-08

**Authors:** Jing Sun, Chi Zhang, Yifang Wang, Siqi Xiao, Haiju Sun, Zhiyuan Bian, Zui Shen, Xiaofen He, Jianqiao Fang, Xiaomei Shao

**Affiliations:** Key Laboratory of Acupuncture and Neurology of Zhejiang Province, The Third Affiliated Hospital of Zhejiang Chinese Medical University, Hangzhou, China

**Keywords:** Pain memory, Rostral anterior cingulate cortex, GABAergic neurons, GABA receptors, Electroacupuncture

## Abstract

Recent studies have confirmed that pain memory is often accompanied by negative emotions. Electroacupuncture (EA) can block the retrieval of painful memories, thereby alleviating the associated negative behaviors. However, the underlying mechanism is poorly understood. This study revealed that the effect of EA on pain memory-induced negative behaviors is related to the mediation of GABAergic neuron activity and GABA receptor expression in the rostral anterior cingulate cortex (rACC). Previous studies have shown that the rACC is a crucial area for regulating nociceptive behaviors and negative emotions in pain memory models. The GABAergic neurons and receptors in the rACC are largely involved in pain sensation and related effects. However, the relationships among pain memory, GABAergic neurons and receptors in the rACC have not been investigated. In this study, we established a pain memory model via secondary plantar cross-injection of carrageenan and EA treatment. Using chemogenetic methods and behavioral assessments of pain and negative emotion, we found that early excitation of GABAergic neurons in the rACC blocked the recall of pain memories and reduced anxiety-like behaviors in pain memory model rats. Furthermore, pharmacological methods revealed that excitation of GABA_A_ and GABA_B_ receptors in the rACC blocks hyperpathia associated with pain memory and pain-induced anxiety-like behaviors, while inhibition of GABA_A_ and GABA_B_ receptors reverses these effects. These results suggest that EA may alleviate pain and associated anxiety-like behaviors related to pain memories through the activation of GABAergic neurons and excitation of GABA_A_ and GABA_B_ receptors in the rACC.

**Supplementary Information:**

The online version contains supplementary material available at 10.1007/s12035-024-03986-z.

## Introduction

Chronic pain is a long-standing health issue known to impair patients’ quality of life [1]. Mood disorders are commonly observed in patients with chronic pain [2, 3]; a previous study showed that 42% of patients with chronic pain had a diagnosis of major depression, and 36.5% of these patients were diagnosed with social phobia [2]. These negative emotions may also exacerbate chronic pain, resulting in an intractable condition of comorbid pain in patients with psychiatric disorders [4, 5], which may lead to the development of cognitive disorders associated with chronic pain [6–8]. Many studies have investigated the mechanisms underlying chronic pain [9], such as the “pain memory” mechanism, which is defined as pain and pain-induced anxiety-like behaviors.

The mechanisms underlying the reinforcement of the interaction between pain and negative emotions are complicated. Several regions in the central nervous system are involved in this process; regions in the medial pain system have been extensively investigated due to their crucial roles in supporting the affective component of pain [10]. Our previous studies showed that the rostral anterior cingulate cortex (rACC) is an important area for regulating nociceptive behavior in a rat model of pain memory [7, 11]. Although many studies on the rACC have focused on its role in the negative effects of pain, less is known about its role in pain-related negative emotions [12–14]. Our previous study showed that the rACC-thalamus glutamatergic circuitry regulates chronic inflammatory pain-induced anxiety-like behaviors in mice [15]. Another study showed that rACC deactivation-caudal ACC (cACC) hyperactivation may underlie anxiety-like behaviors in a rat model of chronic facial inflammatory pain [16]. Gamma-aminobutyric acid (GABA) is a major inhibitory neurotransmitter that plays an important role in the pathogenesis of mood disorders and pain conditions [17, 18]. In a clinical study, magnetic resonance spectroscopy (MRS) of patients with chronic pain showed that reduced GABA in the rACC may lead to increased pain intensity and greater catastrophizing [19]. Rodent studies have shown that stimulation of GABAergic interneurons in the rACC can produce antinociceptive effects [20], and GABAergic dysfunction in the rACC is associated with pain-induced aversiveness [21]. The transplants integrated, exerted a GABA_A_ mediated inhibition of host pyramidal cells and blocked gabapentin preference (i.e. relieved ongoing pain) in a conditioned place preference paradigm [21]. These studies demonstrated that GABAergic neurons and receptors in the rACC are largely involved in pain sensation and related effects, but less is known about the role of these neurons and receptors in pain memory.

Electroacupuncture (EA) has been used in the management of chronic pain conditions and psychiatric disorders [22–24]. Interestingly, a potential positive effect of EA on comorbid pain and psychiatric disorders was demonstrated in a clinical study, which showed that better analgesic effects are achieved with EA in chronic pain patients with higher anxiety levels [25]. On the basis of our previous finding of anxiety-like behaviors in a rat model of pain memory in an open field test, we detected a significant decrease in the duration and distance traveled in the central area after the second carrageenan injection, and nociceptive and anxiety-like behaviors were alleviated by EA treatment [26]. However, the mechanisms underlying these effects remain unclear.

Given that GABAergic neurons in the rACC are involved in mediating both pain sensation and related effects, in the present study, we examined the effects of GABAergic neuron activation and GABA receptor excitation associated with pain in the rACC on the recall of pain memories and related anxiety-like behaviors. We subsequently explored whether the effects of EA on pain memory-induced negative behaviors are related to mediating the activity of GABAergic neurons and GABA receptors in the rACC.

## Materials and Methods

### Experimental Design

The study was divided into three parts. In experiment 1, to determine the activation state of GABAergic neurons in the rACC of pain memory model rats, chemogenetic technology was used to explore whether the activation/inhibition of GABAergic neurons in the rACC could block the recall of pain memory and alleviate hyperalgesia and anxiety-like behaviors in pain memory model rats. In experiment 2, to investigate which GABA receptors are involved in alleviating hyperalgesia and anxiety-like behaviors in the pain memory model based on the results of experiment 1, pharmacological methods were used to explore whether the excitation of GABA_A_ and GABA_B_ receptors in the rACC can alleviate hyperalgesia and anxiety-like behaviors in pain memory model rats. In experiment 3, to elucidate the ability of EA to relieve hyperpathia and anxiety-like behaviors induced by pain memories, chemogenetic and pharmacological methods were used to observe whether the inhibition of GABAergic neurons and GABA_A_ and GABA_B_ receptors in the rACC could block the effect of EA.

### Animals and Groups

A total of 130 adult male Sprague‒Dawley (SD) rats (300 ± 20 g, aged 7–8 weeks) were acquired from the Laboratory Animal Center of Zhejiang Chinese Medical University; these rats were donated by the Association for Assessment and Accreditation of Laboratory Animal Care (AAALAC) and housed four per cage. The rats were maintained on clean corn cob bedding and had free access to rodent chow and water. The living conditions were maintained at 24 ± 1 °C under a 12-hour light/dark cycle (lights on at 7:00 a.m., lights off at 20:00). All procedures were carried out under the guidelines of the National Institutes of Health Principles of Laboratory Animal Care (NIH Publications No. 8,023, revised 1978) and were approved by the Animal Ethics Committee of Zhejiang Chinese Medical University (ZSLL, 2017 − 183).

Healthy male SD rats were randomly divided into the following groups: C + mCherry group, M + mCherry group, M + 3D group, and M + 4D group for experiment 1; M + vehicle group, M + AR agonist group, and M + BR agonist group for experiment 2; and M + mCherry group, M + 3D group, M + EA group, M + EA + 4D group, M + vehicle group, M + EA + vehicle group, M + EA + AR antagonist group, and M + EA + BR antagonist group for experiment 3. The details are shown in Table 1.

**Table 1:** The number of animals and treatment details for each experimental group

Experiment	Group	Number of animals	Description of the treatments
Experiment 1	C + mCherry group	8	Control rats with AAV-VGAT-mCherry injection
	M + mCherry group	8	Model rats with AAV-VGAT-mCherry injection
	M + 3D group	8	Model rats with AAV-VGAT-hM3D-mCherry injection
	M + 4D group	8	Model rats with AAV-VGAT-hM4D-mCherry injection
Experiment 2	M + vehicle group	8	Model rats with vehicle injection
	M + AR agonist group	8	Model rats with GABA_A_ receptor agonist injection
	M + BR agonist group	8	Model rats with GABA_B_ receptor agonist injection
Experiment 3	M + mCherry group	10	Model rats with AAV-VGAT-mCherry injection
	M + 3D group	10	Model rats with AAV-VGAT-hM3D-mCherry injection
	M + EA group	10	Model rats with EA treatment
	M + EA + 4D group	11	Model rats with EA treatment and AAV-VGAT-hM4D-mCherry injection
	M + vehicle group	8	Model rats with vehicle injection
	M + EA + vehicle group	9	Model rats with EA treatment and vehicle injection
	M + EA + AR antagonist group	8	Model rats with EA treatment and GABA_A_ receptor antagonist injection
	M + EA + BR antagonist group	8	Model rats with EA treatment and GABA_B_ receptor antagonist injection

### Carr-Induced Pain Memory Rat Model

We established a pain memory model by injecting carrageenan twice into the paw [27, 6, 7]. On day 1, 100 µl of 2% carrageenan (Carr, 22049-5G-F; Sigma‒Aldrich, USA) was injected into the left hind paw. On day 13, the nociceptive pain induced by the first injection was reversed, and then a second Carr injection into the right hind paw at the same dose was given on day 14. Rats in the C + mCherry group were injected with 100 µL of normal saline at the same time.

### Virus Injection and Guide Cannula Implantation

All surgeries were performed under aseptic conditions and stereotaxic guidance. The rats were intraperitoneally anesthetized with 1% pentobarbital sodium after their heads were shaved by an electric razor, after which the mice were fixed onto a stereotaxic instrument (68,025, RWD, Shenzhen, China). The X and Y coordinate positions are reported with respect to the bregma in mm. The position of the anterior cingulate cortex was determined according to Paxinos and Watson’s Rat Brain Atlas (6th edition) as follows: +2.76 mm anteroposterior (AP), ± 0.8 mm mediolateral (ML), and 1.3 mm dorsoventral (DV).

The virus (AAV-VGAT-hM3D/hM4D-mCherry or AAV-VGAT-mCherry, 450 nl/side, BrainVTA, Wuhan, China) was injected using a 10 µl infusion pump (WPI, UMC4, Sarasota, FL, United States) connected to a microinjector pump (D105114, RWD, Shenzhen, China) at a rate of 40 nl/min. Afterward, the needle was left in place for an additional 8 min to allow the virus to spread at the injection site, after which the needle was slowly withdrawn. For the rats in the M + 3D group, the virus was injected into the hemispheres. All rats in the M + 4D group were bilaterally injected because the unilateral loss of function may be compensated by the other hemisphere.

For microinjection of drugs into the rACC, a cannula (62,203, RWD, China; length C = 0 mm; G = 0.4 mm) was stereotaxically implanted into the rACC at the proper depth under isoflurane (output concentration 2%, oxygen flow 500 ml/min) anesthesia. The cannula was left in place for 5 min, after which the cannula was fixed to the skull with bone screws, super glue, and dental cement. Then, a dummy cannula was inserted into the guide cannula.

### Drug Delivery

For the GABA receptor agonist/antagonist infusion, the solution consisted of the GABA_A_ receptor agonist muscimol (25 nl/µl, GA23204, GLPBIO, China), the GABA_A_ receptor antagonist bicuculline (200 ng/µl, GN10745, GLPBIO, China), the GABA_B_ receptor agonist baclofen (50 nl/µl, GC12927, GLPBIO, China) and the GABA_B_ receptor antagonist CGP55845 (22 nl/µl, 1248, Tocris, China). The infusion cannulas (62,203, RWD, China; length C = 0 mm; G = 0.4 mm) were connected via polyethylene tubing (62,302, RWD, China) to 10 µl microsyringes (Hamilton, Reno, NV) mounted on a microinfusion pump (RWD200, China). To allow diffusion of the drug, the infusion cannulas were kept in place for 5 min before being replaced with dummy cannulas. On days 0 ~ 5, 500 nl of the drug was infused via the infusion cannulas at a flow rate of 100 nl/min each time. The animals were injected without anesthesia at 4.5 h, 1 day, 2 days, 3 days, 4 days, or 5 days after the first carrageenan injection [28].

### EA Treatment

The rats in the M + EA, M + EA + 4D, M + EA + vehicle, M + EA + AR antagonist and M + EA + BR antagonist groups in experiment 3 were subjected to EA treatment from day 0 (after Carr injection for 5 h) to day 5 without anesthesia. EA was administered as previously described [6, 7]. We selected the bilateral Zusanli acupoint (ST36), which is approximately 5 mm lateral to the anterior tubercle of the tibia. We inserted stainless steel acupuncture needles (0.18 mm*13 mm) into the bilateral ST36 region at a depth of 5 mm. The needles were connected to the HANS-200 A acupoint nerve stimulator (Huawei Co., Ltd., Beijing, China). The EA parameters were set as follows: 2/100 Hz, dilatational wave, and intensities ranging from 1 to 2 mA (increased by 0.5 mA every 10 min for 30 min). The animals wore black hoods over their heads for a calming effect during the administration of EA. No signs of stress were observed. To maintain consistency, the rats in the other groups also wore black hoods at the same time.

### Paw Withdrawal Thresholds (PWTs)

The paw withdrawal threshold (PWT) was a nociceptive indicator used to evaluate the pain response in this study. The measurement procedures were performed as described in previous studies [6, 7, 28]. We used a dynamic plantar aesthesiometer (model 37,450; Ugo Basile, Comerio, Italy) to test the PWTs. All rats were placed on wire entanglements using transparent plastic chambers positioned on a wire mesh grid for 30 min for acclimation. An increasing vertical force (increased steadily from 0 to 50 g over 20 s) was applied using a stainless steel probe (a straight 0.5 mm diameter shaft) placed underneath the mesh floor to stimulate the left hind paws. We considered paw withdrawal or licking to indicate a positive response. The PWT was the average of five measurements. PWTs were measured on day − 1 (baseline), day 0 (after Carr injection for 4 h), day 3, day 5, day 13, day 15, and day 17.

### Behavioral Assessment of Negative Emotion

#### Open Field Test (OFT)

The open field chamber (100 × 100 cm) was made of plastic and divided into a central field (central region, 50 × 50 cm) and an outer field (peripheral region). The rats were carefully placed in the center of the open field arena. The more time that the rats spent in the central region, the less likely anxiety-like behaviors were observed. Individual rats were placed in the center field at the start of the test. After 30 s of adaptation, the activity of the rats was recorded for 5 min. The time spent in the central region was recorded to evaluate anxiety-like behaviors [28, 45].

#### Elevated Zero Maze (EZM) Test

The elevated zero maze (EZM) utilized in this study was constructed from a black metal ring platform and measured 1 m in diameter. Positioned 60 cm above the ground, the platform included a 20 cm wide ring, which was further divided into open arms and closed arms. The open arms lacked any form of shielding, while the closed arms were equipped with a baffle measuring 30 cm in height. During the experiment, the rats were positioned between the open arm and the closed arm, with their heads oriented toward the side of the closed arm. The testing procedure for each rat consisted of a 30-second acclimatization period followed by a 5-min test session. The time spent in the open arms was recorded to evaluate anxiety-like behaviors [28, 45].

After each test was performed, the experimenter picked up any feces, cleaned the test area thoroughly with a cloth containing 75% alcohol and water, and finally dried the area with a separate cloth before performing the next experiment. The OFT and EZM sessions were recorded by a video camera. An ANY-maze V6.14 analysis system (Stoelting, IL, USA) was used to track the location, velocity, and movement of the head, body, and tail. All the measurements displayed are relative to the rat body.

### Immunohistochemistry

Three rats in each group were intraperitoneally anesthetized with 1% pentobarbital sodium. Next, the sections were perfused with 0.9% saline, followed by 4% paraformaldehyde. The brains were removed and immediately placed in 4% paraformaldehyde at 4 °C overnight. Afterward, all the brains were dehydrated with 15% and 30% sucrose until they sank. We cut the brains into coronal sections of 30 μm by using a cryostat frozen microtome (Thermo Fisher Scientific, NX50, United States). First, the sections were warmed in a water bath at 37 °C for 1 h. The sections were then washed five times with phosphate-buffered saline (PBS) supplemented with Tween (PBST) on a shaker (10 min each). We incubated the sections with 10% donkey blocking buffer in a water bath at 37 °C for 1 h. Later, the sections were incubated with primary antibodies, including anti-GABA (1:500, rabbit, GTX125988, United States) and anti-c-Fos (1:1000, rabbit, ab190289, Abcam, United States), at 4 °C overnight. Subsequently, the sections were warmed in a 37 °C water bath for 1 h. We washed the sections with PBST five times and then incubated them with secondary antibodies (Alexa Fluor 488-conjugated, 1:800; Jackson ImmunoResearch, United States) in a 37 °C water bath for 1 h. After washing five times with PBST, all the sections were incubated with DAPI (ab104139; Abcam, United States). The images were obtained with a digital pathological section scanner (VS120-S6-W, Olympus).

### Statistical Analysis

The experimental data are expressed as the mean ± standard error (x ± S.E.M.), and all the statistical analyses of the experimental data were performed using SPSS software. Differences in PWTs among multiple groups were analyzed by two-way ANOVA followed by Tukey’s post hoc test. The OFT and EZM data were analyzed using one-way ANOVA followed by an LSD post hoc test. *p* < 0.05 was considered to indicate statistical significance.

## Results

### Early Activation of GABAergic Neurons in the rACC Blocks the Recall of Pain Memory

**Fig. 1:**
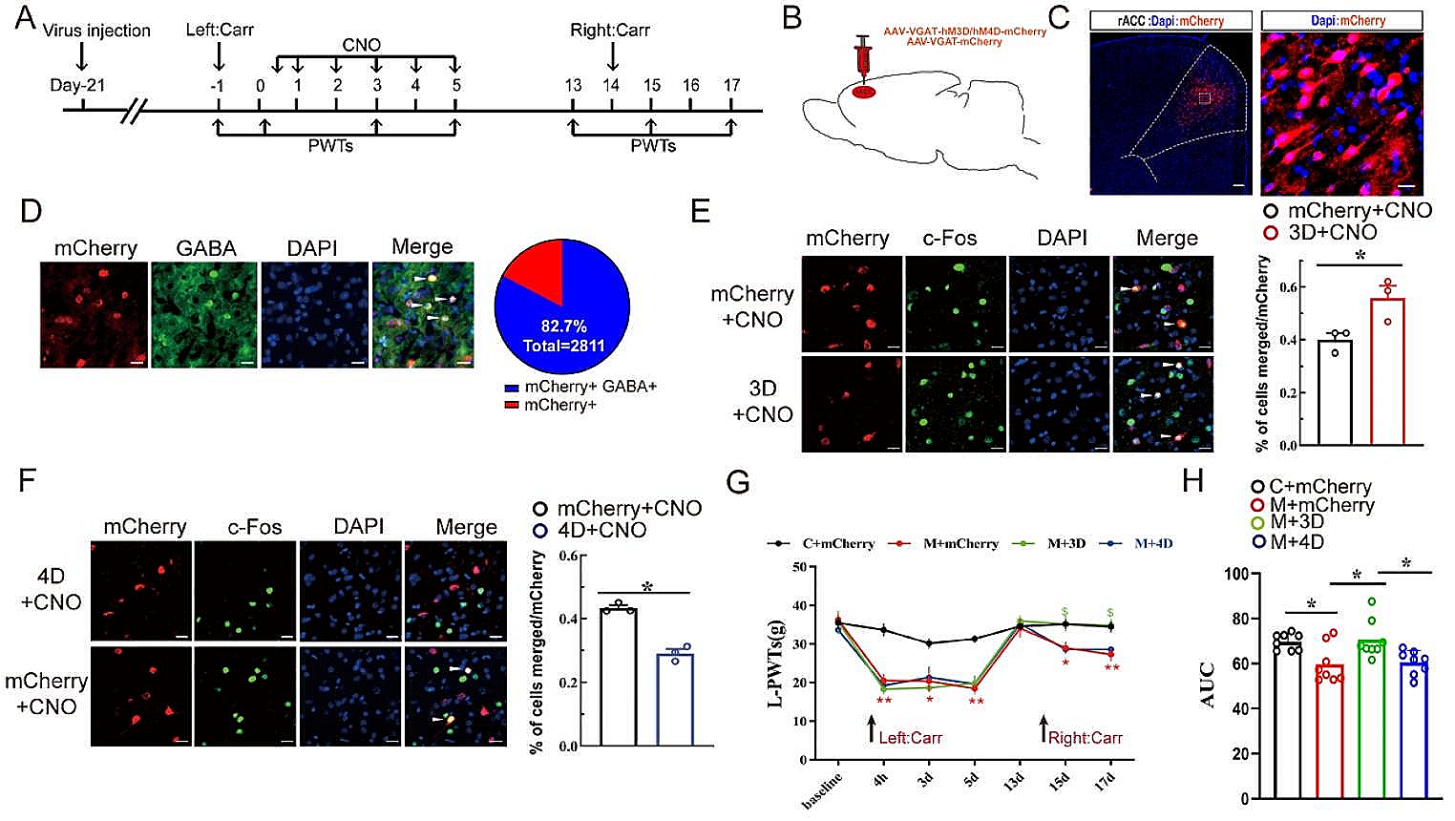
GABAergic neurons in the rACC controlled the PWT in pain memory model rats. **A** Flow chart of the experiment. **B** Location of virus injection in a sagittal view of the rat rACC (AAV-VGAT-hM3D/hM4D-mCherry or AAV-VGAT-mCherry). **C** Representative images of virus expression in the rACC after virus injection (scale bar: 200 μm/20 µm). **D** Representative images of the colocalization of mCherry (red) and GABAergic neuron markers (green) in the rACC (scale bars: 20 μm) (left) and the percentage of mCherry and GABAergic neuron colocalization (*n* = 3 rats) (right). **E/F** Efficacy tests of the hM3Dq and hM4Di viruses. Representative images of mCherry (red) merged with c-Fos (green) in the rACC (scale bars: 20 μm) (left) and the percentage of colocalization between mCherry and c-Fos (*P < 0.05) (*n* = 3 rats). **G** Changes in paw withdrawal thresholds (PWTs) in each group (*P < 0.05, **P < 0.01 * C + mCherry group vs. M + mCherry group. $*P* < 0.05, $ M + mCherry group vs. M + 3D group.) (*n* = 8 rats). **H** Comparison of the area under the curve (AUC) of the PWT on days 13–17 in each group (*P < 0.05) (*n* = 8 rats)

We established a rat model of pain memory via the injection of 2% carrageenan into the left hind paw on day 0 and the same dose of Carr into the right hind paw on day 14 (Fig. 1A). Histological validation of the control and model groups revealed that rACC GABAergic neuronal activity differed before and after Carr treatment. Compared with that in the control group, the rACC GABAergic neuronal activity in the model group was significantly lower [t test: t = 3.421, *P* = 0.0019] (Figure S1).

On day − 21, we injected adeno-associated virus (AAV-VGAT-hM3Dq/hM4Di-mCherry or AAV-VGAT-mCherry) into the rACC to specifically manipulate GABAergic neuron activity in the rACC (Fig. 1A-B). Then, we detected the fluorescence of the virus in the rACC (Fig. 1C). The results showed that 82.7% of mCherry colocalized with the GABAergic neurons (Fig. 1D). The excitatory role of the hM3D/hM4D virus was determined by comparing the colocalization percentage of the virus-labeled neurons with c-Fos between the control mCherry group and the hM3D/hM4D virus group (Fig. 1E-F). The above results showed that the virus significantly activated or inhibited GABAergic neurons in the rACC. On days 15–17, the PWTs of the rats in the M + mCherry group were significantly lower than those of the rats in the C + mCherry group, and the PWTs of the rats in the M + 3D group were significantly greater than those of the rats in the M + mCherry group [two-way rmANOVA: group × time: F (18, 196) = 4.017, *P* < 0.0001; time: F (6, 196) = 48.56, *P* < 0.0001; group: F (3, 196) = 26.09, *P* < 0.0001; followed by Tukey post hoc test: *P* < 0.0001, *P* < 0.0001] (Fig. 1G). On days 13–17, compared with those of the rats in the M + mCherry group, the AUCs of the PWTs of the rats in the C + mCherry group and the M + 3D group both increased [one-way ANOVA: F (3, 28) = 5.995, *P* = 0.0027; followed by the LSD post hoc test: *P* < 0.05, *P* < 0.05]. The AUCs of the PWTs of the rats in the M + 3D group increased more than those of the rats in the M + 4D group [LSD post hoc test: *P* < 0.05, *P* < 0.05] (Fig. 1H). Taken together, our data indicate that early activation of GABAergic neurons in the rACC could block the recall of pain memories, but early inhibition of GABAergic neurons in the rACC had no effect on hyperpathia.

### Activation of GABAergic Neurons in the rACC Reduces Anxiety-Like Behaviors in Pain Memory Model Rats

**Fig. 2:**
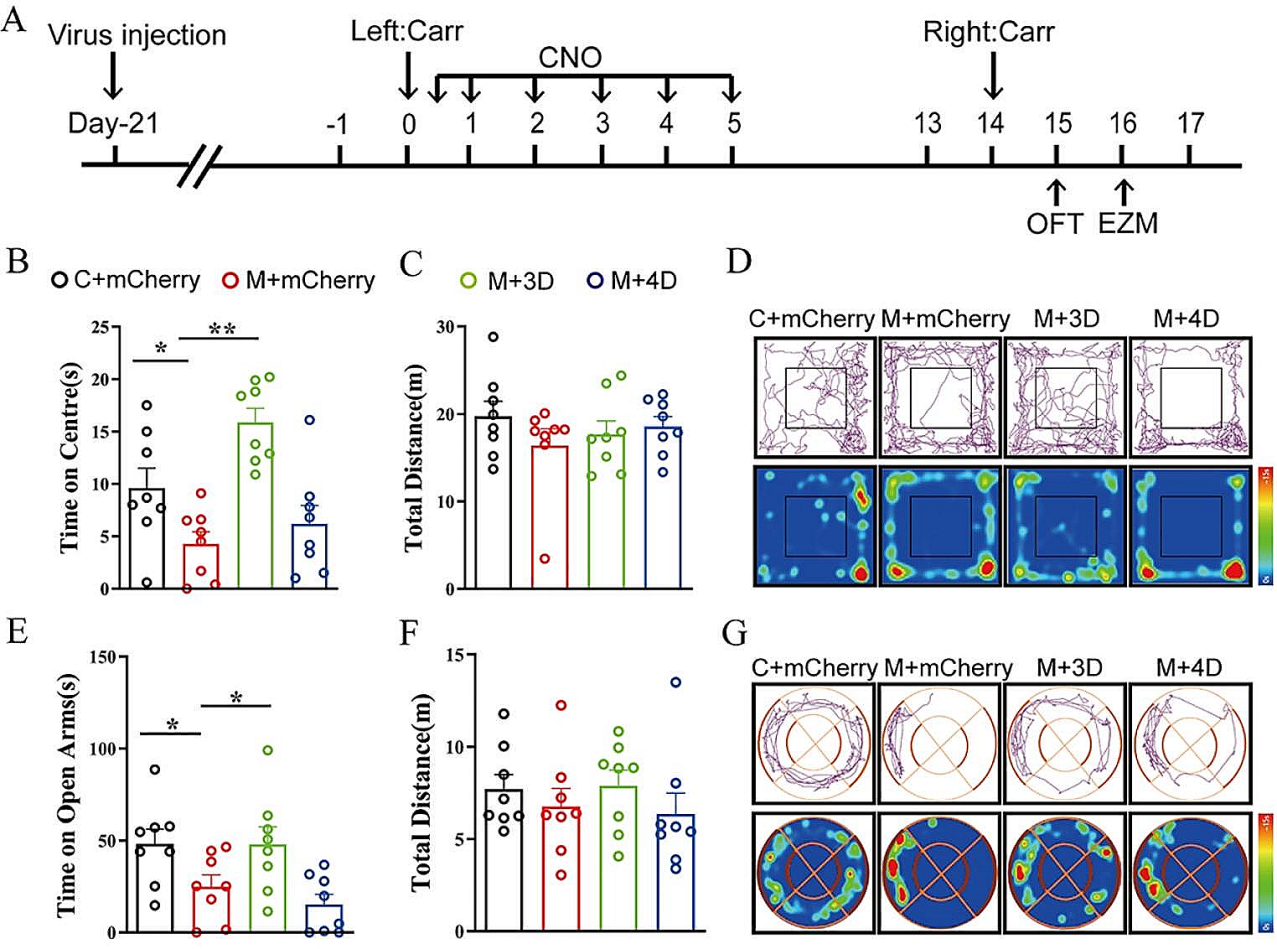
GABAergic neurons in the rACC modulate pain memory-induced anxiety-like behaviors. **A** Flow chart of the experiment. **B** The time spent in the center in the OFT by each group (*P < 0.05, **P < 0.01) (*n* = 8 rats). **C** The total distance the rats traveled in the OFT (*n* = 8 rats). **D** Representative movement trajectories and heatmaps for each group in the OFT. **E** The time that the rats spent in the open arms in the EZM for each group (*P < 0.05, **P < 0.01) (*n* = 8 rats). **F** The total distance traveled by the rats in the EZM (*n* = 8 rats). **G** Representative movement trajectories and heatmaps of the EZM test for each group

To test our hypothesis, we used behavioral tests (OFT and EZM test) combined with a chemogenetic manipulation strategy. After the second Carr injection, the anxiety-like behaviors of the rats were assessed through the OFT and EZM test (Fig. 2A). Compared with the rats in the C + mCherry group, the rats in the M + mCherry group spent less time in the central area in the OFT [one-way ANOVA: *F*_(3,28)_ = 10.66, *P* < 0.0001; followed by the LSD post hoc test: *P* < 0.0001, *P* < 0.0001]. The rats in the M + 3D group spent more time in the center in the OFT than the rats in the M + mCherry group [LSD post hoc test: *P* < 0.0001, *P* < 0.0001] (Fig. 2B). There was no significant difference in the total distance traveled during the OFT between the two groups [one-way ANOVA: *F*_(3,28)_ = 0.7993, *P* = 0.5497] (Fig. 2C). In the EZM test, the rats in the C + mCherry group spent more time in the open arms than the rats in the M + mCherry group [one-way ANOVA: *F*_(3,28)_ = 4.963, *P* = 0.0069; followed by the LSD post hoc test: *P* < 0.05, *P* < 0.05]. The rats in the M + 3D group spent more time in the open arms than the rats in the M + mCherry group [LSD post hoc test: *P* < 0.05, *P* < 0.05] (Fig. 2E). The total distance traveled in the EZM was not significantly different among the rat groups [one-way ANOVA: *F*_(3, 28)_ = 0.5980, *P* = 0.6216] (Fig. 2F). Therefore, these results suggest that early activation of GABAergic neuron activity in the rACC reduces anxiety-like behaviors in pain memory model rats.

### The Excitation of GABA _A_ and GABA_B_ Receptors in the rACC Blocks the Recall of Hyperpathia Induced by a Second Cross-Carr Injection

**Fig. 3:**
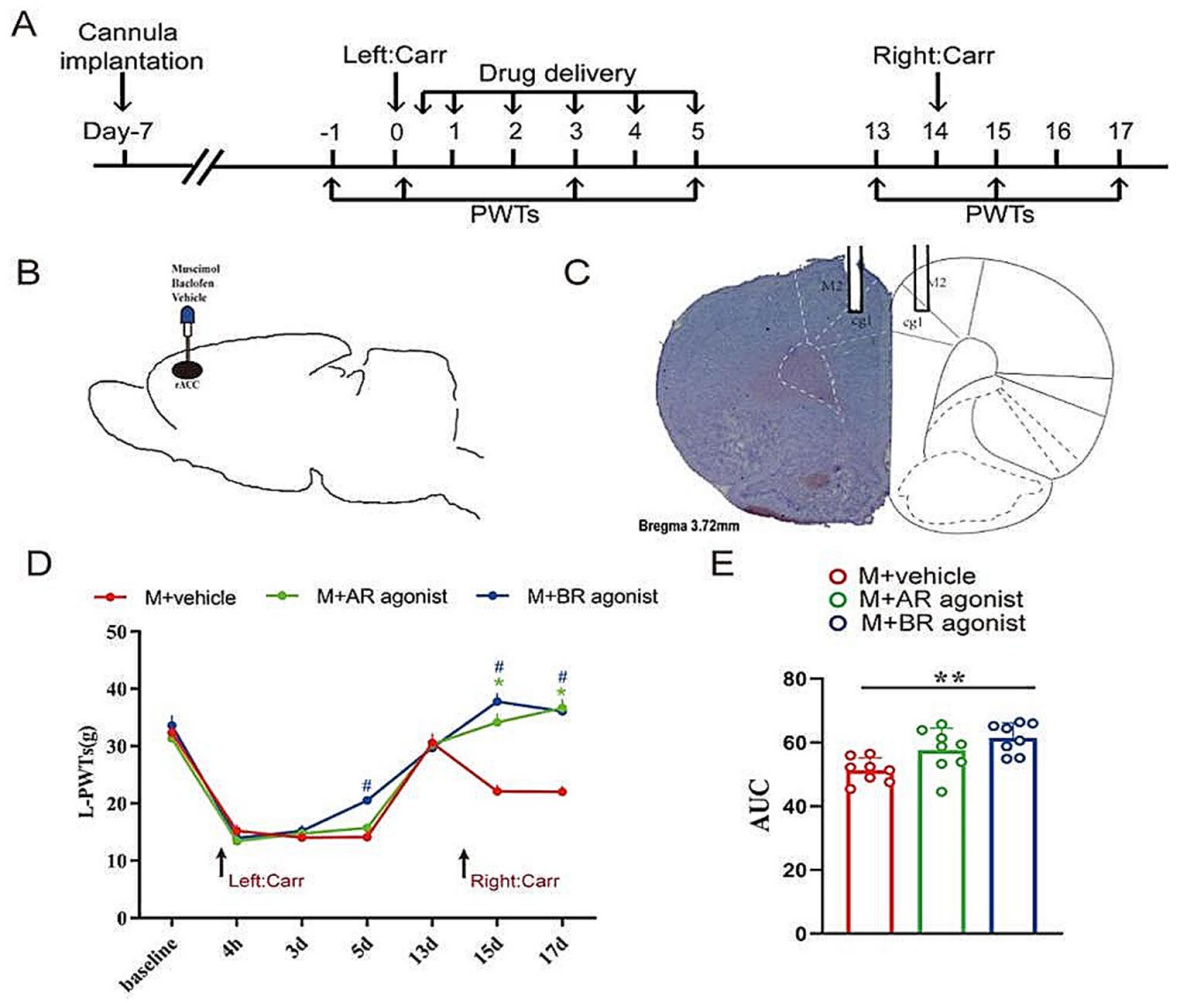
Excitation of GABA_A_ and GABA_B_ receptors in the rACC relieves hyperpathia in pain memory model rats. **A** Flow chart of the experiment. **B** The location of the implanted cannula in the sagittal view. **C** Representative Nissl staining diagram showing the location of the cannula in the rACC in coronal sections (right). **D** Changes in PWTs in each group (*P < 0.05, * M + vehicle group vs. M + AR agonist group). ^#^*P* < 0.05, ^#^ M + vehicle group vs. M + BR agonist group) (*n* = 8 rats). (E) Comparison of the PWT AUCs on days 13–17 in each group (**P < 0.01) (*n* = 8 rats)

Subsequently, we aimed to determine whether GABA_A_ and GABA_B_ receptor excitation in the rACC had similar effects in model rats. First, we implanted a cannula in the rACC one week before the first Carr injection (Fig. 3A). We confirmed the correct location in the rACC through Nissl staining (Fig. 3B-C). Next, GABA_A_ and GABA_B_ receptor agonists were delivered into the rACC on days 0 ~ 5 (Fig. 3A). Additionally, we tested the rats’ PWTs on certain days. On day 17, compared with those of the rats in the M + vehicle group, the PWTs of the rats in the M + AR agonist group and the M + BR agonist group were significantly greater after the second Carr injection [two-way rmANOVA: group × time: *F*_(12, 147)_ *=* 11.29, *P* < 0.0001; time: *F*_(6, 147)_ = 155.0, *P* < 0.0001; group: *F*_(2, 147)_ *=* 34.17, *P* < 0.0001; followed by Tukey post hoc test: *P* < 0.0001, *P* < 0.0001] (Fig. 3D). On days 13–17, the AUCs of the PWTs of the rats in the M + BR agonist group were greater than those of the of the rats in the M + vehicle group (one-way ANOVA: *F*_(2, 21)_ = 7.519, *P* = 0.0034), followed by the LSD post hoc test: *P* < 0.05, *P* < 0.05) (Fig. 3E). Therefore, our results indicated that exciting GABA_A_ and GABA_B_ receptors in the rACC can relieve hyperpathia in pain memory model rats after a second Carr injection.

### The Excitation of GABA_A_ and GABA_B_ Receptors in the rACC Blocks Pain-Induced Anxiety-Like Behaviors

**Fig. 4:**
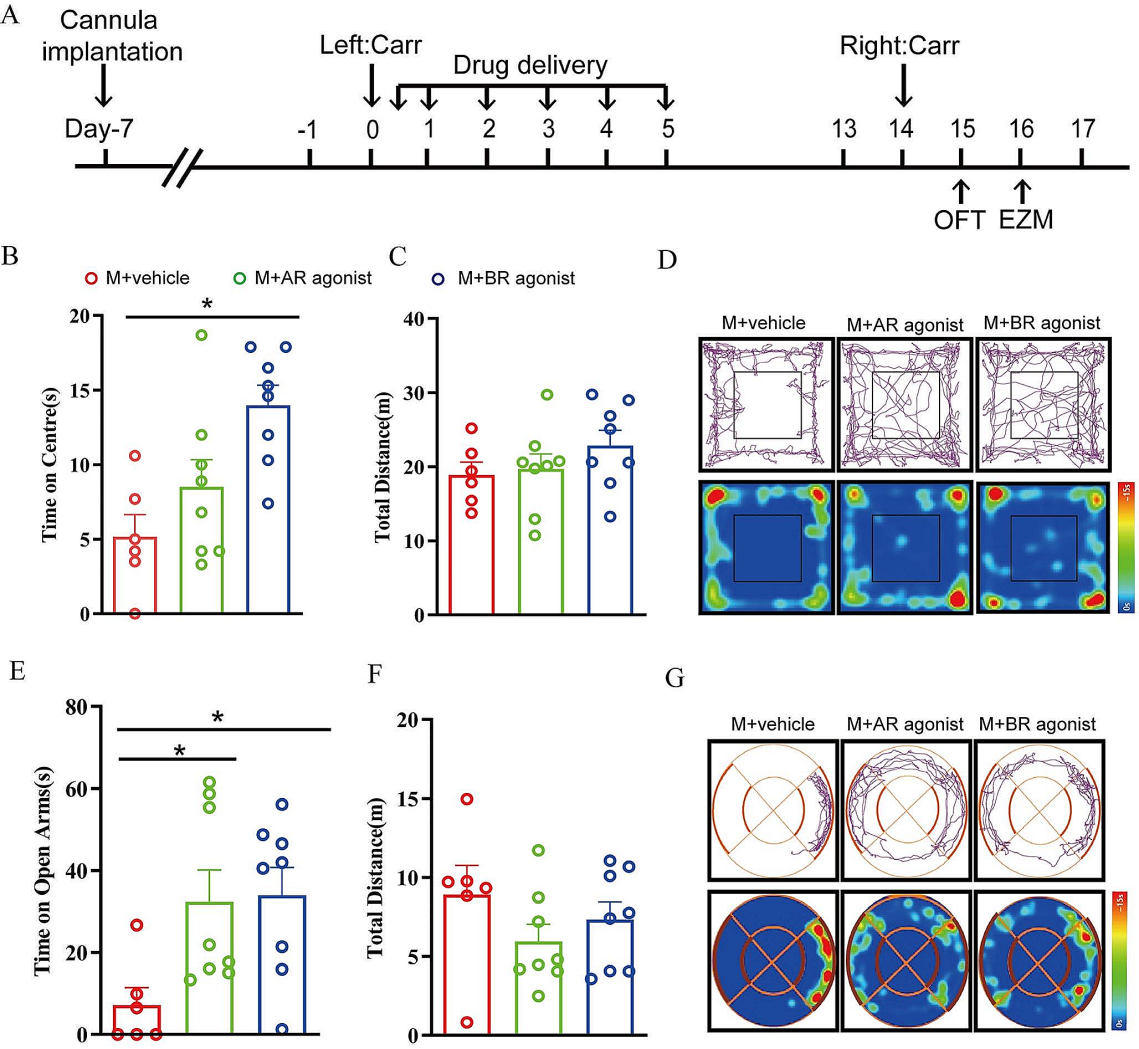
The excitation of GABA_A_ and GABA_B_ receptors in the rACC can block the anxiety-like behaviors induced by pain memory. **A** Flow chart of the experiment. **B** The time spent in the center in the OFT in each group (*P < 0.05) (*n* = 6/8 rats). **C** The total distance traveled by the rats in the OFT (*n* = 6/8 rats). **D** Representative movement trajectories and heatmaps in the OFT for each group. **E** The time that the rats spent in the open arms in the EZM for each group (*P < 0.05) (*n* = 6/8 rats). **F** The total distance traveled by the rats in the EZM (*n* = 6/8 rats). **G** Representative movement trajectories and heatmaps in the EZM for each group

To investigate the role of GABA_A_ and GABA_B_ receptors in anxiety-like behaviors in model rats, we administered the same drug to manipulate the activity of GABA_A_ and GABA_B_ receptors in the rACC (Fig. 4A). Then, we examined anxiety-like behaviors through the OFT and EZM test. Compared with the rats in the M + vehicle group, the rats in the M + BR agonist group spent more time in the central area in the OFT [one-way ANOVA: *F*_(2, 19)_ = 7.595, *P* = 0.0038; followed by the LSD post hoc test: *P* < 0.05, *P* < 0.05]; however, the central time in the M + AR agonist group did not decrease compared with that in the M + vehicle group [the LSD post hoc test: *P* < 0.05, *P* < 0.05] (Fig. 4B). There was no significant difference in the total distance traveled in the OFT among the rats in the three groups [one-way ANOVA: *F*_(2, 19)_ *=* 1.101, *P =* 0.3528] (Fig. 4C). According to the EZM test, the rats in the M + AR agonist group and the M + BR agonist group spent more time in the open arms than the rats in the M + vehicle group [one-way ANOVA: *F*_(2, 19)_ *=* 4.409, *P =* 0.0267; followed by the LSD post hoc test: *P* < 0.05, *P* < 0.05] (Fig. 4E); and the total distance traveled in the EZM test was not significantly different among the groups [one-way ANOVA: *F*_(2, 19)_ *=* 1.201, *P =* 0.3229] (Fig. 4F). Taken together, these results suggest that the excitation of GABA_A_ and GABA_B_ receptors in the rACC relieves pain-induced anxiety-like behaviors and that the effect of GABA_B_ receptors is superior to that of GABA_A_ receptors.

### Inhibition of GABAergic Neurons in the rACC Reverses the Therapeutic Effect of Electroacupuncture on Hyperpathia Induced by Pain Memory

**Fig. 5:**
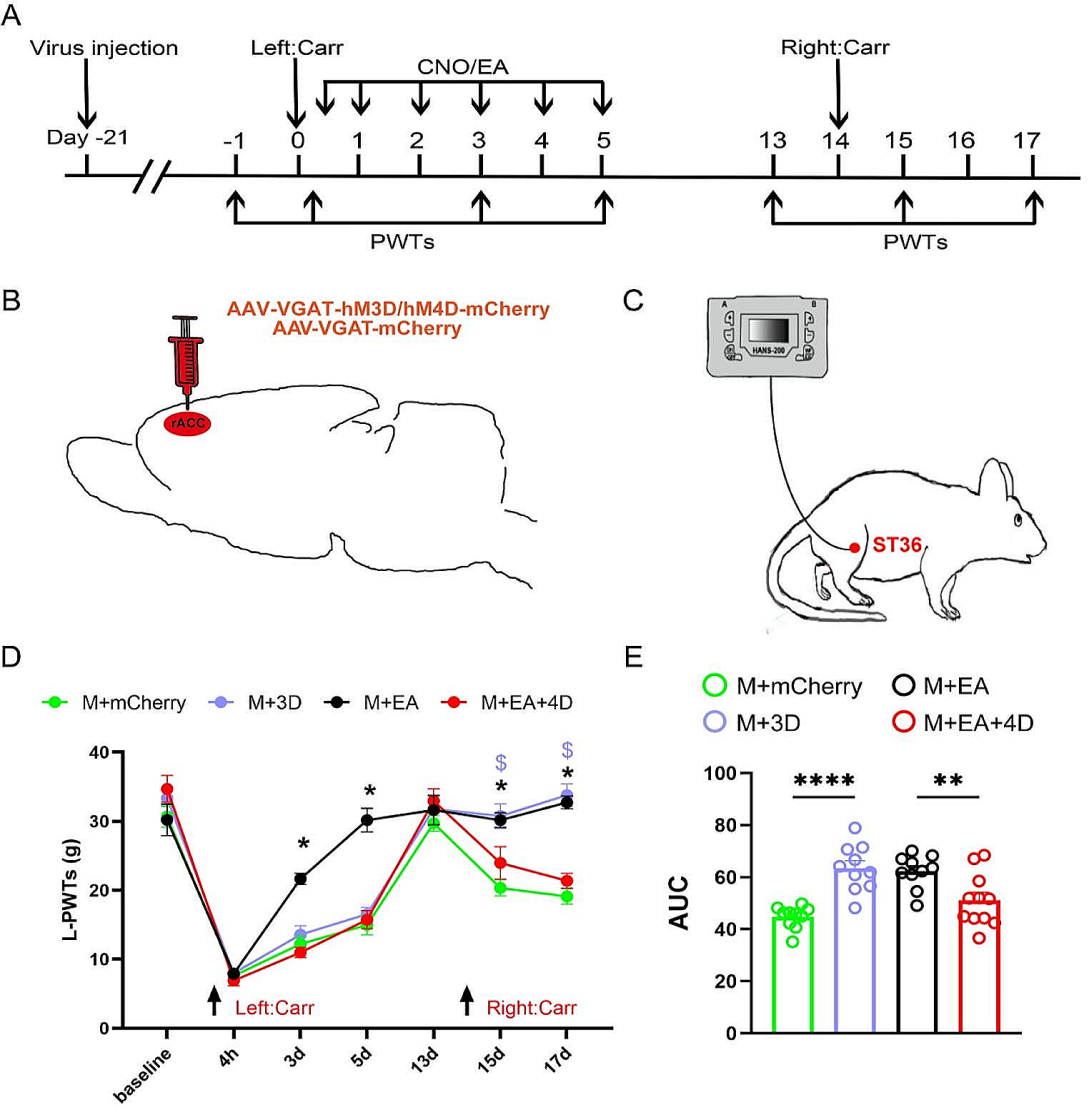
The inhibition of GABAergic activity in the rACC can block the ability of EA to relieve hyperpathia induced by pain memory. **A** Flow chart of the experiment. **B** Location of virus injection in a sagittal view of the rat rACC (AAV-VGAT-hM3D/hM4D-mCherry or AAV-VGAT-mCherry). **C** Schematic diagram of EA treatment in ST36. **D** Changes in PWTs in each group (*P < 0.05, * M + mCherry group vs. M + 3D group). ^$^*P* < 0.05, ^$^ M + EA group vs. M + EA + 4D group) (*n* = 10/11 rats). **E** Comparison of PWT AUCs on days 13–17 in each group (**P < 0.01, ****P < 0.0001) (*n* = 10/11 rats)

We subsequently investigated the correlation between EA and GABAergic neuron activity in the rACC during hyperpathia-induced pain memory. On day − 21, we injected adeno-associated viruses (AAV-VGAT-hM3Dq/hM4Di-mCherry or AAV-VGAT-mCherry) into the rACC to specifically manipulate GABAergic neuron activity in the rACC. After the first Carr injection, we administered intraperitoneal CNO and EA treatment on days 0 ~ 5 (Fig. 5A-C). The EA stimulation was administered in the bilateral ST36 region (Fig. 5C). On day 17, compared with those of the rats in the M + mCherry group, the PWTs of the rats in the M + 3D group were significantly greater; moreover, compared with those of the rats in the M + EA group, those of the rats in the M + EA + 4D group were significantly lower [two-way rmANOVA: group × time: *F*_(18, 259)_ = 7.729, *P* < 0.00 501; time: *F*_(6, 259)_ = 165.1, *P* < 0.0001; group: *F*_(3, 259)_ = 34.03, *P* < 0.0001; followed by Tukey post hoc test: *P* < 0.0001, *P* < 0.0001] (Fig. 5D). On days 13–17, the AUCs of the PWTs of the rats in the M + mCherry group was lower than those of the rats in the M + 3D group; moreover, the AUCs of the PWTs of the rats in the M + EA group were greater than those of the rats in the M + EA + 4D group [one-way ANOVA: *F*_(3, 37)_ = 13.39, *P* < 0.0001; followed by the LSD post hoc test: *P* < 0.0001, *P* < 0.05] (Fig. 3E). Taken together, the above results indicate that inhibition of GABAergic neurons in the rACC reverses the therapeutic effect of EA on hyperpathia induced by pain memory.

### Inhibition of GABAergic Neurons in the rACC Reverses the Therapeutic Effect of Electroacupuncture on Anxiety-Like Behaviors Induced by Pain Memory

**Fig. 6:**
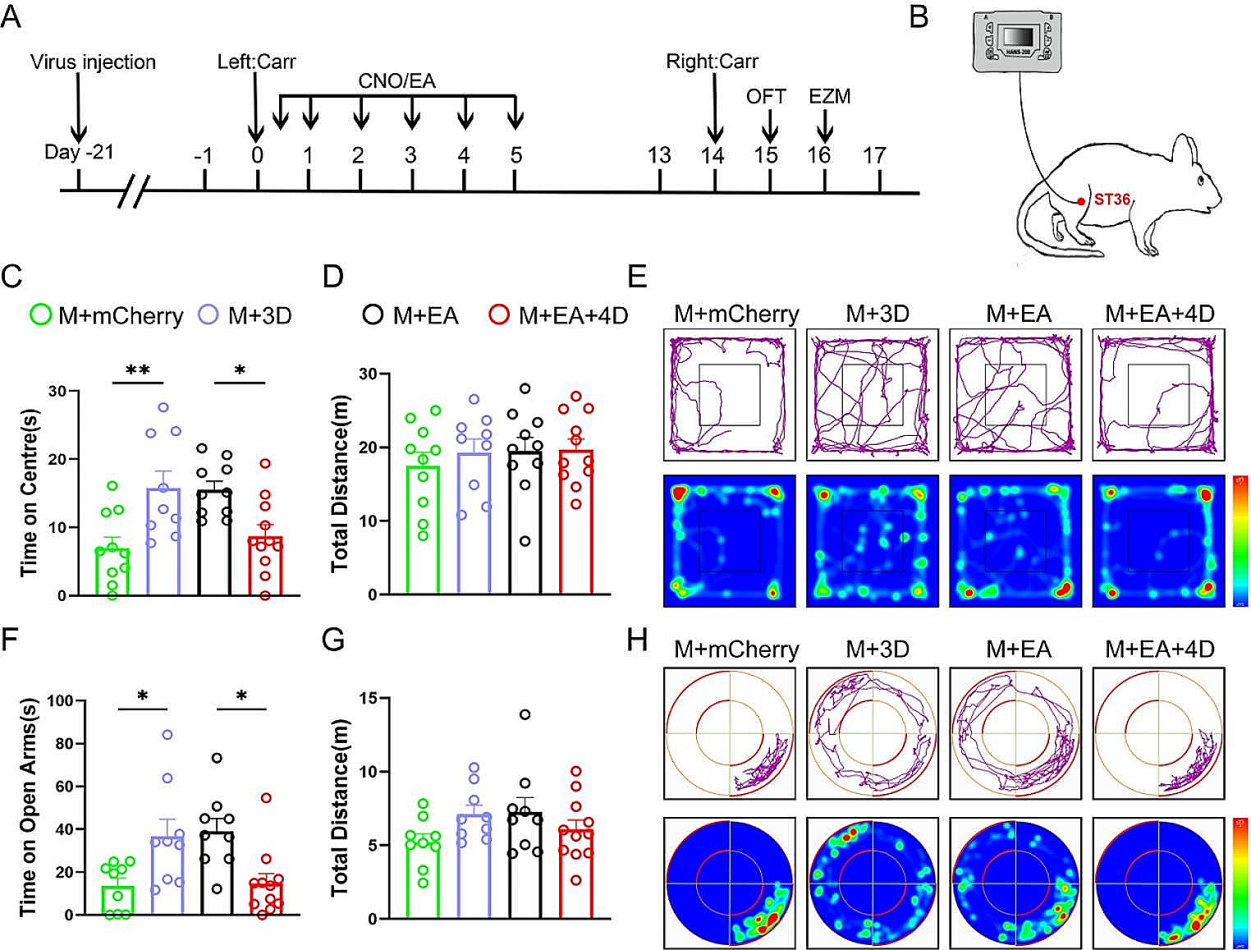
The inhibition of GABAergic activity in the rACC can block the ameliorative effect of EA on anxiety-like behaviors induced by pain memory. **A** Flow chart of the experiment. **B** Schematic diagram of EA treatment in ST36. **C** The time spent in the center in the OFT in each group (*P < 0.05, **P < 0.01) (*n* = 9/11 rats). **D** The total distance traveled by the rats in the OFT (*n* = 9/11 rats). **E** Representative movement trajectories and heatmaps in the OFT for each group. **F** The time that the rats spent in the open arms in the EZM in each group (*P < 0.05) (*n* = 9/11 rats). **G** The total distance traveled by the rats in the EZM (*n* = 9/11 rats). **H** Representative movement trajectories and heatmaps in the EZM for each group

We next investigated whether the inhibition of GABAergic neurons in the rACC could reverse the anxiolytic effects of EA on pain memory-induced anxiety-like behaviors. First, we injected adeno-associated viruses (AAV-VGAT-hM3Dq/hM4Di-mCherry or AAV-VGAT-mCherry) into the rACC to specifically manipulate GABAergic neuron activity in the rACC. Second, we intraperitoneally administered CNO and EA on days 0 ~ 5 after the first Carr injection. Then, we assessed anxiety-like behavior in the OFT and EZM following secondary Carr injection (Fig. 6A). The EA stimulation was administered in the bilateral ST36 region (Fig. 6B). Compared with those in the M + mCherry group, the rats in the M + 3D group spent more time in the central area in the OFT; moreover, compared with those in the M + EA group, the rats in the M + EA + 4D group spent less time in the central area in the OFT [one-way ANOVA: *F*_(3, 36)_ = 6.500, *P* = 0.0013; followed by the LSD post hoc test: *P* < 0.01, *P* < 0.05] (Fig. 6C). The total distance traveled by the rats in the OFT did not significantly differ among the four groups [one-way ANOVA: *F*_(3, 36)_ *=* 0.3562, *P =* 0.7849] (Fig. 6D). In the EZM test, the rats in the M + 3D group spent more time in the open arms than the rats in the M + mCherry group; moreover, the rats in the M + EA + 4D group spent less time in the open arms than the rats in the M + EA group [one-way ANOVA: *F*_(3, 34)_ *=* 5.942, *P =* 0.0023; followed by the LSD post hoc test: *P* < 0.05, *P* < 0.05] (Fig. 6F). Furthermore, the total distance traveled by the rats in the EZM test was not significantly different among the groups [one-way ANOVA: *F*_(3, 34)_ *=* 1.735, *P =* 0.1782] (Fig. 6G). Overall, our data showed that inhibition of GABAergic neurons in the rACC reverses the anxiolytic effects of EA on pain memory-induced anxiety-like behaviors.

### Inhibition of GABA_A_ and GABA_B_ Receptors in the rACC Reverses the Therapeutic Effect of Electroacupuncture on Hyperpathia Induced by Pain Memory

**Fig. 7:**
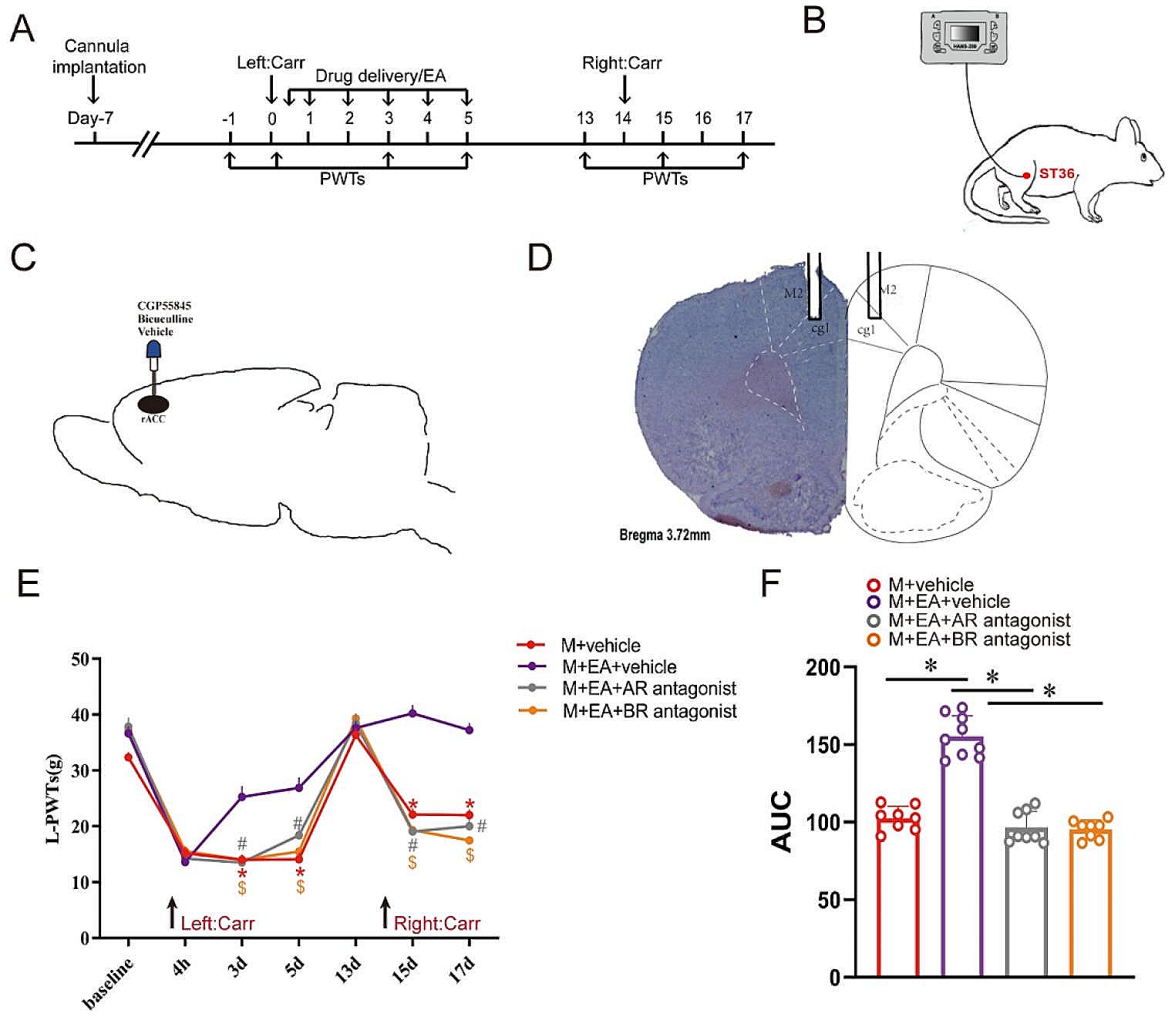
Inhibition of GABA_A_ and GABA_B_ receptors in the rACC blocks the ability of EA to relieve hyperpathia induced by pain memory. **A** Flow chart of the experiment. **B** Schematic diagram of EA treatment in ST36. **C** The location of the implanted cannula in a sagittal view. **D** Representative Nissl staining diagram showing the location of the cannula in the rACC in a coronal section (right). **E** Changes in PWT after treatment in each group (*P < 0.05, * M + EA + vehicle group vs. M + vehicle group). ^#^*P* < 0.05, ^#^ M + EA + vehicle group vs. M + AR agonist group. ^$^*P* < 0.05, M + EA + vehicle group vs. M + BR agonist group) (*n* = 8/9 rats). **F** Comparison of the PWT AUCs on days 13–17 in each group (*P < 0.05) (*n* = 8/9 rats)

We next assessed the association between EA and GABA_A_ and GABA_B_ receptor activity in the rACC during hyperpathia-induced pain memory. After the first Carr injection, we delivered GABA_A_ and GABA_B_ receptor antagonists to the rACC during EA treatment on days 0 ~ 5 (Fig. 7A). The EA stimulation was administered in the bilateral ST36 region (Fig. 7B). The location of the implanted cannula in the rACC was confirmed by Nissl staining (Fig. 7C-D). Compared with those of the rats in the M + EA + vehicle group, the PWTs of the rats in the M + vehicle group, the M + EA + AR antagonist group, and the M + EA + BR antagonist group were significantly lower [two-way rmANOVA: group × time: *F*_(18, 203)_ = 13.50, *P* < 0.0001; time: *F*_(6, 203)_ = 196.9, *P* < 0.0001; group: *F*_(3, 203)_ = 77.22, *P* < 0.0001; followed by Tukey post hoc test: *P* < 0.0001, *P* < 0.0001] (Fig. 7E). Compared with those of the rats in the M + EA + vehicle group on days 13–17, the area under the curve (AUC) of the rats in the M + vehicle group, the M + EA + AR antagonist group, and the M + EA + BR antagonist group were significantly lower [one-way ANOVA: *F*_(3, 29)_ = 74.08, *P* < 0.0001; followed by the LSD post hoc test: *P* < 0.0001, *P* < 0.0001] (Fig. 7F). These results proved that EA can relieve hyperpathia induced by pain memory and that these mechanisms may occur through the activation of GABA_A_ and GABA_B_ receptors.

### Inhibition of GABA_A_ and GABA_B_ Receptors in the rACC Reverses the Therapeutic Effect of Electroacupuncture on Anxiety-Like Behaviors Induced by Pain Memory

**Fig. 8:**
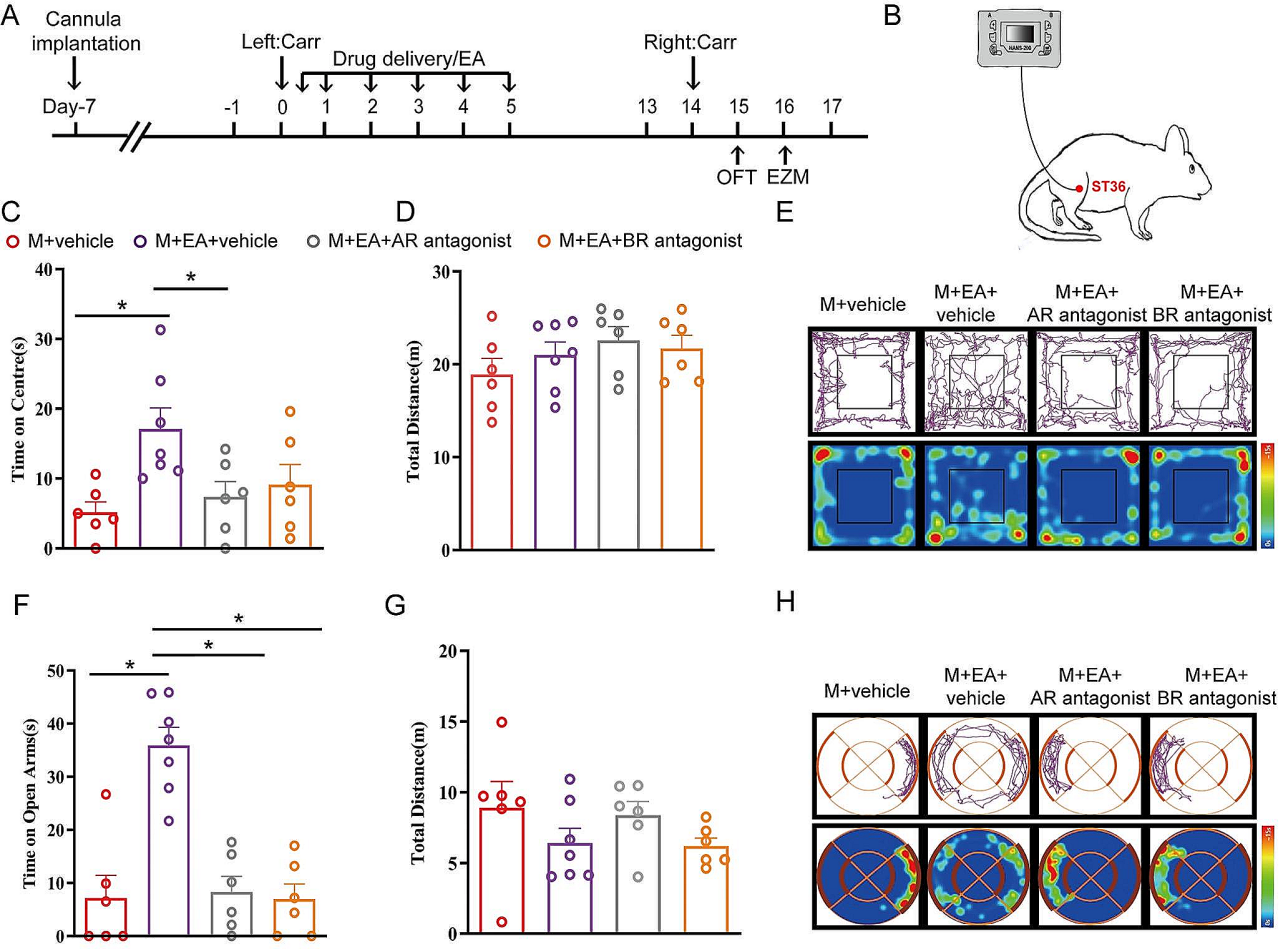
The inhibition of GABA_A_ and GABA_B_ receptors in the rACC can reverse the effect of EA on relieving anxiety-like behaviors induced by pain memory. **A** Timeline for drug delivery and EA treatment. **B** Schematic diagram of EA treatment in ST36 cells. **C** The time spent in the center in the OFT in each group (*P < 0.05) (*n* = 6/8 rats). **D** The total distance traveled by the rats in the OFT (*n* = 6/7 rats). **E** Representative locomotor trajectories and heatmaps of each group of rats in the OF test. **F** The time that the rats spent in the open arms in the EZM in each group (*P < 0.05) (*n* = 6/7 rats). **G** The total distance traveled by the rats in the EZM (*n* = 6/7 rats). **H** Example of locomotor trajectories and heatmaps for each group in the EZM test

We next examined whether inhibiting GABA_A_ and GABA_B_ receptors in the rACC could reverse the effect of EA on anxiety-like behaviors induced by pain memory. First, rats were injected with GABA_A_ and GABA_B_ receptor antagonists in the rACC and subjected to EA treatment on days 0 ~ 5 (Fig. 8A-B). The rats were subsequently assigned to groups according to their anxiety-like behaviors in the OFT and EZM test on days 15 and 16, respectively (Fig. 8A). In the OFT, compared with those in the M + vehicle group, the rats in the M + EA + vehicle group spent more time in the central area (*P < 0.05) (Fig. 8C). Compared with those in the M + EA + vehicle group, the rats in the M + EA + AR group and the M + EA + BR group spent less time in the central area in the OFT [one-way ANOVA: *F*_(3, 21)_ *=* 4.554, *P =* 0.0131; followed by the LSD post hoc test: *P* < 0.05, *P* < 0.05] (Fig. 8C). There was no significant difference in the total distance traveled by the rats among the groups [one-way ANOVA: *F*_(3, 21)_ = 1.047, *P* = 0.3923] (Fig. 8D). Compared with those in the M + EA + vehicle group, the rats in the M + EA + AR group and the M + EA + BR group spent less time in the open arm in the EZM [one-way ANOVA: *F*_(3, 21)_ *=* 18.49, *P <* 0.0001; followed by the LSD post hoc test: *P <* 0.0001, *P <* 0.0001] (Fig. 8F). The total distance traveled by the rats was not significantly different among the groups [one-way ANOVA: *F*_(3, 21)_ *=* 1.304, *P* = 0.2995] (Fig. 8G). Hence, our results showed that EA relieves the anxiety-like behaviors induced by pain memory through excitation of the GABA_A_ and GABA_B_ receptors.

## Discussion

The main objective of this study was to explore the associations among pain memory, GABAergic neurons, GABA_A_ receptors and GABA_B_ receptors in the rACC. Activation of GABAergic neurons and GABA_A_ and GABA_B_ receptors in the rACC relieved pain and pain-induced anxiety-like behaviors in pain memory model rats. Subsequently, the effects of EA intervention and GABA_A_ and GABA_B_ receptor antagonism were investigated. EA intervention had a similar effect on the activation of GABAergic neurons. When GABA_A_ and GABA_B_ receptors were antagonized, the effect of EA was reversed. The results suggest that the effect of EA intervention on pain memory in chronic pain patients may occur through the excitation of GABA_A_ and GABA_B_ receptors in the rACC.

Previous studies have shown that in a rat model of repeated acute injuries caused by 2 crossover carrageenan injections, the initial injury-induced allodynia in the injured hindpaw reoccurs after the same injury is given to the previously uninjured hindpaw [29, 30]. Thus, an indelible “pain memory” of the initial injury persists, even after the recovery of the PWT of the injured hindpaw. Thus, pain memory may underlie the development of chronic pain. Some evidence has shown that the nociceptive response to the inflammatory process associated with carrageenin does not vary with age but rather depends on the patient’s pain history, which is related to memory mechanisms [31]. However, the underlying mechanism of pain memory and ways to relieve pain memory are not yet fully understood.

GABA is the main inhibitory neurotransmitter in the central nervous system. The common receptors of GABA associated with pain include GABA_A_ and GABA_B_. GABA receptor dysfunction is linked to a wide variety of disorders, including anxiety, depression, alcohol addiction, memory disorders, and cancer [32]. Previous studies have shown that GABA_A_ receptors play different roles in acute and chronic pain [33]. During acute pain, the selective activation of GABA_A_ receptor subtypes may reverse the loss of postsynaptic inhibition mediated by GABA_A_ receptors in the spinal cord, which could result in analgesic effects. It has also been reported that during chronic pain, drugs that negatively regulate GABA_A_ receptors, which could change presynaptic inhibitory neurotransmission, may also be effective analgesics [33]. GABA_B_ receptors are G protein-coupled receptors (GPCRs) that mediate slow and prolonged inhibitory neurotransmission in the brain and are associated with several behavioral diseases, including epilepsy, spasticity, anxiety, and pain [34]. There is substantial evidence that GABA_B_ receptors are involved in the processing of pain signals and thus have long been considered valuable targets for the generation of analgesics for treating chronic pain [35]. A previous study showed that activating GABA_B_ receptors could promote the fading of fear memories [36]. GABA_A_ receptors are rapid-acting, ligand-gated ion channel (LGIC) receptors. This type of receptor may have antinociceptive and pronociceptive effects in healthy and chronic pain-affected animals, respectively, indicating its viability as a valid pharmacological target for treating chronic pain [37]. A previous study indicated that peripheral GABA_A_ receptors and endogenous GABA, possibly produced by inflamed tissue, potentiate CFA-induced persistent inflammatory hypersensitivity, suggesting that they can be used as therapeutic targets for alleviating inflammatory pain [38]. A pain memory model of injury-induced allodynia was established with carrageenan in our study. Therefore, both GABA_A_ and GABA_B_ receptors are activated in response to pain memory.

The ACC is a major target in the medial pain pathway. It plays an important role in the affective component related to pain perception and memory processing. Previous evidence has shown that the ACC is important in the acquisition of nociception-related memories [39]. This area is heterogeneous, and its subdivisions, i.e., the rACC and caudal ACC (cACC), contribute differently to pain processing. Moreover, previous studies have shown that, in a rat model, activation of the rACC, but not the cACC, is necessary to induce pain-related aversion using a formalin-induced conditioned place aversion paradigm [12, 40]. Bilateral lesions in the rACC did not change formalin-induced nociceptive behaviors but reduced pain-related aversion [41]. In a rat model of neuropathic pain, activation of GABA receptors in the rACC did not change mechanical hyperalgesia but reduced pain-related aversion [42]. These results indicate that the rACC is specifically involved in the affective component of pain but not in the sensory component. Similarly, in the present study, the results showed that the excitation of GABAergic neurons in the rACC did not change the sensory hypersensitivity induced by the first carrageenan injection. However, this excitation successfully prevented the secondary hyperalgesia induced by the second carrageenan injection into the previously noninjected hindpaw. The mechanisms of secondary hyperalgesia are poorly understood, but the rACC is likely involved in this process. Another study demonstrated that a cross-callosal neural circuit in the rACC contributes to contralateral secondary hyperalgesia, which was also observed in mice subjected to CCI, and the inhibition of cross-callosal projection neurons in the rACC contralateral to the injured side selectively normalized secondary hyperalgesia in the uninjured limb without changing the nociceptive sensation of the primary injury [43]. Our results indicate that GABAergic neurons in the rACC also mediate contralateral secondary hyperalgesia, but we do not know whether GABAergic neurons affect only contralateral secondary hyperalgesia or whether GABAergic neurons are involved in a cross-callosal neural circuit.

The analgesic and antidepressive effects of EA have been investigated extensively. EA can reduce sensory hypersensitivity and pain-related negative emotions in both inflammatory [15, 44] and neuropathic pain models [45, 46]. Consistent with our previous findings [46], EA reduced primary hyperalgesia after the first carrageenan injection, and this early EA intervention blocked the recall of pain memories induced by the second carrageenan injection. The present study also showed that EA can reduce anxiety-like behaviors in a pain memory model. Previously, we revealed the functional significance of GABA_B_ receptors in the EA-mediated attenuation of pain memories and related anxiety-like behaviors in the midcingulate cortices of rats [47]. In the present study, we observed that the inhibition of GABAergic neurons and GABA_A_ and GABA_B_ receptors in the rACC reversed the therapeutic effect of EA. Our results indicated that the activation of GABAergic neurons and excitation of GABA_A_ and GABA_B_ receptors in the rACC may be associated with the effect of EA on relieving pain memory-related behaviors.

## Conclusion

In conclusion, we demonstrated that the activation of GABAergic neurons and GABA_A_ and GABA_B_ receptors in the rACC can relieve pain and pain-induced anxiety-like behaviors related to pain memory in chronic pain patients. Furthermore, EA may alleviate pain and pain-induced anxiety-like behaviors associated with pain memory in chronic pain patients through the activation of GABAergic neurons and excitation of GABA_A_ and GABA_B_ receptors in the rACC.

### Electronic Supplementary Material

Below is the link to the electronic supplementary material.

Supplementary Material 1

Supplementary Material 2

Supplementary Material 3

## Data Availability

The original data presented in the study will be made available by the authors without undue reservation.
