# Supplementary material for: Electroacupuncture Alleviates Hyperalgesia and Anxiety-Like Behaviors in Pain Memory Model Rats Through Activation of GABAergic Neurons and GABA Receptor in the Rostral Anterior Cingulate Cortex
Source: Mol Neurobiol. 2024 Feb 8;61(9):6613–27. doi: 10.1007/s12035-024-03986-z (PMC11338974; doi:10.1007/s12035-024-03986-z)
Supplement: Supplementary file 1 — Supplementary Material 1 [file 12035_2024_3986_MOESM1_ESM.pdf]

Molecular Neurobiology

Supplementary Information

Electroacupuncture alleviates hyperalgesia and anxiety-like behaviors in pain memory model rats through activation of GABAergic neurons and GABA receptor in the rostral anterior cingulate cortex

Jing Sun[1†], Chi Zhang[1†], Yifang Wang[1†], Siqi Xiao[1], Haiju Sun[1], Zhiyuan Bian[1], Zui Shen[1], Xiaofen He[1], Jianqiao Fang[1*] and Xiaomei Shao[1*]

[1] Key Laboratory of Acupuncture and Neurology of Zhejiang Province, The Third Affiliated Hospital of Zhejiang Chinese Medical University, Hangzhou, China

*Corresponding authors:

Jianqiao Fang

fangjianqiao7532@163.com

Xiaomei Shao

13185097375@163.com

†Jing Sun, Chi Zhang and Yifang Wang contributed equally to this work.

Supplemental Methods

Immunohistochemistry

The 3 rats of each group were intraperitoneally anesthetized with 1% pentobarbital sodium. Next, they were perfused with 0.9% saline, followed by 4% paraformaldehyde. The brains were removed and immediately placed in 4% paraformaldehyde at 4°C overnight. After that, all brains were dehydrated by 15% and 30% sucrose until they sank. We cut the brains into coronal sections of 20 μm by using a cryostat frozen microtome (Thermo Fisher Scientific, NX50, United States). First, the sections were rewarmed at 37°C in a water bath for 1 h. They were then washed with TBST three times on a shaker (10 min each time). We used 10% donkey-blocking buffer to incubate the sections at 37°C in a water bath for 1 h. Later, the sections were incubated with primary antibodies, including anti-GABA (1:500, rabbit, GTX125988, United States) and anti-c-Fos (1:800, rat, sysy226, Synaptic Systems, United States), at

4°C overnight. Subsequently, they were rewarmed in a 37°C water bath for 1 h. We washed them with TBST three times, then incubated them with secondary antibodies (Alexa Fluor 488-AffiniPure Donkey Anti-Rabbit IgG, 1:500, Jackson Immunoresearch, United States and AlexaFlour 647-AffiniPure Donkey Anti-Rat IgG, 1:500, Jackson Immunoresearch, United States) in a 37°C water bath for 1 h. After washing six times with TBST, all sections were incubated with DAPI (ab104139, Abcam, United States). The images were scanned by a digital pathological section scanner (Axioscan 7, ZEISS).

Supplemental Results:

The co-localization of GABAergic neurons and c-Fos in the rACC of the rat pain memory model was different before and after Carr treatment.

We established a rat model of pain memory via injection of 2% carrageenan into the left hind paw at day 0 and the same dose of Carr into the right hind paw on day 14 (Figure 1A). The experiments were followed by histological validation. Histological validation of the control and model groups showed that rACC GABAergic neuronal activity was different in the two groups. The rACC GABAergic neuronal activity was significantly lower in the model group compared to the control group [t-test: t = 3.421, P = 0.0019]（Figure S1A and B）.

Supplemental Figure:

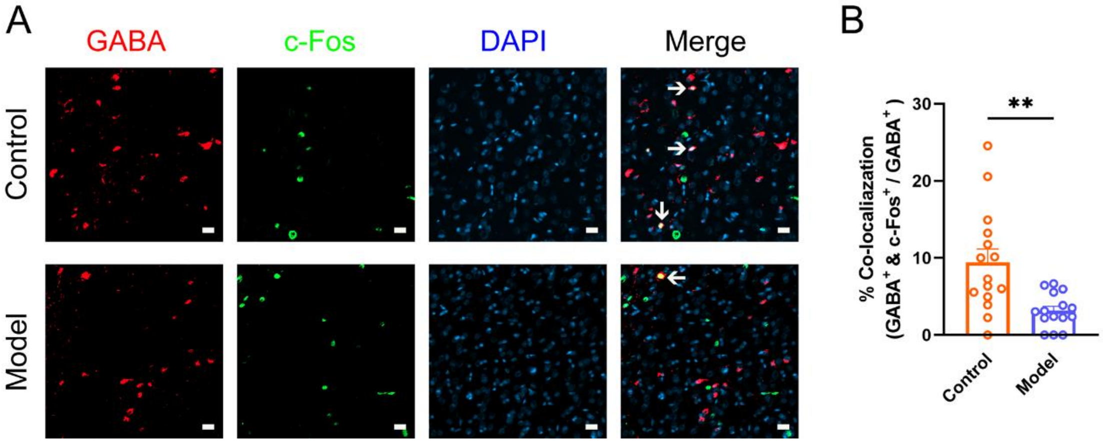

Fig. S1 Immunofluorescence images of c-Fos and GABA co-labelled neurons before and after Carr treatment. (A) Representative images showing GABAergic (red) and c-Fos (green) co-staining in the rACC of the control and model groups. Arrows indicate c-Fos expression in $GABA^+$ neurons, scale bar, 20 μm. (B) Proportion of GABAergic-expressing neurons co-labelled with c-Fos in the rACC (**P < 0.01) (n= 15 sections from three rats).
