# Supplementary material for: Electroacupuncture Alleviates Hyperalgesia and Anxiety-Like Behaviors in Pain Memory Model Rats Through Activation of GABAergic Neurons and GABA Receptor in the Rostral Anterior Cingulate Cortex
Source: Mol Neurobiol. 2024 Feb 8;61(9):6613–27. doi: 10.1007/s12035-024-03986-z (PMC11338974; doi:10.1007/s12035-024-03986-z)
Supplement: Supplementary file 3 — Supplementary Material 3 [file 12035_2024_3986_MOESM3_ESM.pdf]

Editing Certificate

This document certifies that the manuscript

Electroacupuncture alleviates hyperalgesia and anxiety-like behaviors in pain memory model rats through activation of GABAergic neurons and GABA receptor in the rostral anterior cingulate cortex

prepared by the authors

Jing Sun, Chi Zhang, Yifang Wang, Siqi Xiao, Haiju Sun, Zhiyuan Bian, Zui Shen, Xiaofen He, Jianqiao Fang, Xiaomei Shao

was edited for proper English language, grammar, punctuation, spelling, and overall style by one or more of the highly qualified native English speaking editors at AJE.

This certificate was issued on **January 4, 2024** and may be verified on the AJE website using the verification code **D15A-874F-30DE-5B72-DE4P**.

Neither the research content nor the authors' intentions were altered in any way during the editing process. Documents receiving this certification should be English-ready for publication; however, the author has the ability to accept or reject our suggestions and changes. To verify the final AJE edited version, please visit our verification page at aje.com/certificate. If you have any questions or concerns about this edited document, please contact AJE at support@aje.com.

AJE provides a range of editing, translation, and manuscript services for researchers and publishers around the world.
For more information about our company, services, and partner discounts, please visit aje.com.
